# Mendelian susceptibility to mycobacterial diseases: State of the puzzle

**DOI:** 10.5339/qmj.2023.sqac.25

**Published:** 2023-11-26

**Authors:** Abderrahmane Errami, Ahmed Aziz Bousfiha

**Affiliations:** ^1^Laboratory of Clinical Immunology, Inflammation, and Allergy (LICIA), Faculty of Medicine and Pharmacy, Hassan II University, Casablanca, Morocco Email: Erraami@gmail.com ORCID: 0000-0002-2916-5007; ^2^Department of pediatric infectious and immunological diseases, Abderrahim El Harouchi Children Hospital, University Hospital Center Ibn Rochd, Casablanca, Morocco Email: Erraami@gmail.com ORCID: 0000-0002-2916-5007

**Keywords:** Mycobacterial diseases, Monogenic, Inborn errors of immunity, Mendelian susceptibility, IFN-γ

## Abstract

The constant progress of genomics and the establishment of new functional tests have paved the way for identifying monogenic defects conferring a selective predisposition to infections by certain microbes as a new type of inborn errors of immunity (IEIs). Mendelian susceptibility to mycobacterial diseases (MSMD) is the most characterized of these IEIs, with 36 different disorders found in 20 distinct genes (*IFNGR1, IFNGR2, IFNG, IL12RB1, IL12RB2, IL23R, IL12B, ISG15, USP18, ZNFX1, TBX21, STAT1, TYK2, IRF8, IRF1, CYBB, JAK1, RORC, NEMO*, and *SPPL2A*) over the last 20 years. MSMD confers a selective susceptibility to infections with weakly virulent mycobacteria, including the *M*. bovis Bacille Calmette-Guerin (BCG) vaccines and various environmental mycobacteria in patients, primarily children, without classical immune defects. These patients may also present severe forms of tuberculosis, and about half of them might develop non-typhoidal salmonellosis. In some cases, patients also suffer from chronic mucocutaneous candidiasis (CMC), while in others, patients also present severe viral, parasitic, fungal, and/or bacterial diseases. Despite this clinical and genetic heterogeneity, almost all genetic etiologies of MSMD alter the interferon-gamma (IFN-γ)- mediated immunity by impairing or abolishing IFN-γ production or the response to this cytokine. It was proven that the human IFN-γ level is a quantitative trait that defines the outcome of mycobacterial infection. The study of these monogenic defects contributes to understanding the molecular mechanism of mycobacterial diseases in humans and to the development of new diagnostic and therapeutic approaches to improve care and prognosis. For example, MSMD patients with impaired production of IFN-γ may benefit from injections of human recombinant IFN-γ, while for patients with abolished response to this cytokine, hematopoietic stem cell transplantation (HSCT) and promising gene therapy are the only current therapeutic options. These discoveries also bridge the gap between simple Mendelian inheritance and complex human genetics.
